# 4-Hydroxy-2-nonenal causes nuclear accumulation of p62 by inhibiting Xpo1 and promoting the proteolytic pathway in the nucleus

**DOI:** 10.1371/journal.pone.0316558

**Published:** 2025-02-03

**Authors:** Emi Kayama, Ning Baoshuo, Rinna Tatsuno, Kanako Nishi, Elsayed S. I. Mohammed, Yumi Abiko, Toru Yanagawa, Satoru Takahashi, Eiji Warabi

**Affiliations:** 1 Department of Biomedical Science, Institute of Medicine, University of Tsukuba, Tsukuba, Japan; 2 Department of Histology and Cytology, Faculty of Veterinary Medicine, South Valley University, Qena, Egypt; 3 Graduate School of Biomedical Sciences, Nagasaki University, Nagasaki, Japan; 4 Department of Clinical Medicine, Institute of Medicine, University of Tsukuba, Tsukuba, Japan; University of Rijeka Faculty of Medicine: Sveuciliste u Rijeci Medicinski fakultet, CROATIA

## Abstract

p62, an adapter protein involved in selective autophagy, is mainly found in the cytoplasm under normal conditions. Because p62 has nuclear localization signal (NLS) and a nuclear export signal, it has been suggested that p62 shuttles between the nucleus and cytoplasm. We studied the effect of 4-hydroxy-nonenal (4-HNE), an endogenous lipid peroxidation product, on intracellular p62 distribution in mouse embryonic fibroblasts. We found that treatment of 4-HNE causes p62 translocation from the cytoplasm to the nucleus. Further analysis revealed that 4-HNE directly binds to exportin-1 (Xpo1), essential protein for nuclear export of various proteins. Further analysis 4-HNE enhanced intranuclear EGFP-NLS-CL1 degradation in a p62-dependent manner. Our results suggest that 4-HNE changes p62 localization to the nucleus by inhibiting Xpo1 and might affect intranuclear protein quality control.

## Introduction

Unsaturated fatty acids, which are important cell membrane lipid components, undergo oxidative modification, which results in the generation of electrophilic aldehydes, such as 4-hydroxy-2-nonenal (4-HNE) *in vivo*. These electrophilic aldehydes accumulate with age and lead to oxidative stress-related diseases, such as atherosclerosis, diabetes, and neurodegenerative disorders [1–4]. Specifically, 4-HNE is a biomarker of oxidative stress and is considered a potent regulator of many cellular processes, including cell growth, transformation, and death [5, 6].

In cells, various proteins shuttle between the nucleus and cytoplasm [7] owing to the presence of nuclear localization signals (NLS) and nuclear export signals (NES) and the action of the importin complex and exportin1 (Xpo1) [8, 9]. Xpo1 transports more than 100 cargo proteins out of the nucleus by recognizing NES [7, 9]. Leptomycin B (LMB), a known specific inhibitor of Xpo1, forms a complex with Xpo1 by covalently binding to cysteine 528 in the NES-binding region of Xpo1 [10–12]. This binding prevents Xpo1 from binding to cargo proteins and thus inhibits nuclear protein export and results in cargo accumulation in the nucleus. Pankiv et al. reported that p62 has two NLSs (NLS1, 2) and one NES, shuttling between the cytoplasm and nucleus mainly via NLS2 and NES [13].

p62 is a multifunctional protein that is involved in various cellular processes, including selective autophagy, the regulation of transcription factor Nrf2, intracellular aggregate formation, and the formation of liquid droplets via liquid-liquid phase separation, owing to the action of multiple domains [14–18]. Pankiv et al. reported that p62 co-localizes in the nucleus with promyelocytic leukemia bodies (PMLs) and is essential for the accumulation of polyubiquitinated proteins in PML bodies [13]. However, the significance of shuttling and the function of p62 in the nucleus remain unclear.

Recently, it was reported that p62 undergoes liquid-liquid phase separation in the nucleus and forms droplets [19, 20] containing polyubiquitinated proteins, 26S proteasomes, ubiquitinating enzymes E1, E2, and E3, and deubiquitinating enzymes. It has also been shown that p62 contributes to the activation of the nuclear ubiquitin-proteasome system (UPS) [20].

In this study, we aimed to investigate the effect of the electrophilic aldehyde, 4-HNE on intracellular localization of p62 as well as the underlying mechanism using mouse embryonic fibroblasts (MEFs).

## Materials and methods

### Cell culture

MEFs were established from wild-type (WT) or *p62*-deficient (*p62*-KO) mice [16] as previously described [21]. For establishment, animals were anesthetized with isoflurane inhalation and subsequently euthanized by cervical dislocation performed by an experienced individual. The cells were cultured in Dulbecco’s modified Eagle’s medium (DMEM) (Thermo Fisher Scientific) supplemented with 10% fetal bovine serum (FBS), 1% L-glutamine, 1% sodium pyruvate, and 1% penicillin-streptomycin. The cultures were maintained at 37°C in a 5%-CO_2_ environment. The experiments with mice were conducted in accordance with relevant Japanese and institutional regulations and guidelines, with approval from the University of Tsukuba Animal Ethics Committee (authorization number: 23–050). The 10^5^ cells were plated on collagen-coated glass bottom dish (MATSUNAMI) and used for the experiments. 4-HNE (Cayman Cayman Chemical) or leptomycin B (Cayman Chemical) were diluted to the specified concentrations and add to the medium.

### Immunostaining

Cultured cells grown in a 35 mm dish were washed twice with phosphate buffered saline (PBS; Gibco) and fixed with PBS solution containing 4% paraformaldehyde (Nacalai Tesque) at room temperature for 30 minutes. Thereafter, the cells were again washed three times with PBS and treated with PBS containing 0.1% polyoxyethylene octylphenyl ether (Wako) (PBS-Tween20) at room temperature for 30 minutes. Next, the washing solution was removed, and the cells were treated with PBS-Tween20-1% bovine serum albumin (BSA; General Grade, pH 7.0, Nacalai Tesque) at room temperature for 1 hour. Primary antibodies (anti-p62, 1/400) [22] were then added to react with the cells overnight at 4°C. The next day, the cells were washed three times with PBS for 10 minutes. This was followed by incubation with a secondary antibody (horseradish peroxidase (HRP)-conjugated anti-rabbit IgG, 1/800) at room temperature for 1 hour. Finally, after washing three times with PBS for 10 minutes, the cells were observed under a BZ-X810 microscope (Keyence).

### Calculating cytoplasmic-to-nuclear ratio of p62 fluorescence intensity

After immunostaining, the p62 fluorescence intensity was quantified using ImageJ software. The fluorescence intensity was measured for individual cells, with localization facilitated by DAPI staining. Specifically, we quantified the p62 intensity within the nucleus and subtracted this value from the total cellular intensity to obtain the cytoplasmic p62 intensity. A total of 50 cells were analyzed for each experimental group to ensure statistical robustness.

### Construction of vectors

To construct the pEGFP-NLS-CL1 vector, pEGFP-C1 (Clontech) was digested with XhoI (Takara) or PstI (Takara), and the SV-40-derived NLS sequence (cca aaa aag aga aag gta gat cca aaa aag aga aag gta) or the CL1 sequence (gat ccg ctt gta aaa att ggt ttt ctt tat ctc att ttg tta ttc att tat aag), which serves as a proteasome degradation signal, was cloned using the In-Fusion cloning method (Clontech). To generate pEGFP-NLS, we digested pEGFP-NLS-CL1 with HindIII (Takara) or BamHI (Takara) to remove CL1. Thereafter, the pEGFP-NLS obtained was cloned via the ligation method using the Klenow fragment. pEGFP-NLS-NES was then constructed in a similar manner by digesting pEGFP-NLS-CL1 with HindIII or BamHI, amplifying the NES sequence derived from pcDNA3.1-p62 (gct cag tct ctg aca gag caa atg aaa aag ata gcc ttg gag tcg gtg gga cag) using PRIME STAR (Takara), and cloning it using the In-Fusion cloning method (Clontech). Further, a vector with a Lys-to-Ser substitution in the NES region of pEGFP-NLS-NES was generated via mutagenesis (Takara).

### Transfection

The day before gene transfection, the cultured cells were seeded at a density of 10^5^ cells per well onto Poly-Lysine coated 35 mm dishes (Matsunami). The next day, the plasmids were transfected into cells using FuGENE HD (Promega). After 48 hours of transfection, cells were used for subsequent experiments.

### Preparation of recombinant Xpo1

To produce the recombinant Xpo1 protein, NEB Express *E*. *coli* cells (New England BioLabs) containing the pGEX vector (Cytiva) with the Xpo1 gene were added to 2 mL of PlusGRO II medium (Nacalai Tesque) supplemented with ampicillin. This was followed culturing overnight at 37°C after which, 250 mL of LB medium supplemented with ampicillin and the cell culture were transferred to a 500 mL flask, and culturing was continued at 37°C. When the *E*. *coli* cells reached the logarithmic growth phase, IPTG was added to a final concentration of 1 mM, and the culture was again incubated overnight at 30°C. Centrifugation was then performed the next day at 3500 rpm for 10 min at 4°C to collect the *E*. *coli* cells, which were then resuspended in PBS. Further, the suspension solution thus obtained was centrifuged at 3500 rpm and 4°C for 10 minutes after which the *E*. *coli* cells were again resuspended in 20 mL of GST buffer (20 mM Tris-HCl, pH 8.0, 1 mM EDTA, 1% Triton X-100, and protease inhibitors) and sonicated on ice until the solution became translucent. The post-sonication solution was then centrifuged at 1500 rpm for 10 minutes at 4°C and the supernatant was collected as the *E*. *coli* extract. Glutathione-Sepharose 4B (Cytiva) was added to the *E*. *coli* extract (200 μL), and the mixture was incubated overnight at 4°C on a rotator. The next day, the mixture was centrifuged at 6000 rpm for 1 min at 4°C, and the supernatant was removed. The pellet obtained was resuspended in 500 μL of PBS and collected in a 1.5-mL tube. Thereafter, it was washed four times with 1.2 mL of PBS under conditions of 500 *g* for 1 minute at 4°C, followed by washing with PreScission buffer (50 mM Tris-HCl, pH 7.5, 150 mM NaCl, 1 mM EDTA, 1 mM dithiothreitol). Next, the pellet was resuspended in a mixture of 16 μL of PreScission Protease (Cytiva) and 184 μL of PreScission buffer and incubated on a rotator at 4°C for 4 hours. Centrifugation was them performed on the mixture at 500 *g* for 1 minute at 4°C, and the supernatant was collected as the purified protein, which was then stored at -80°C until further analysis.

### Biotin-PEAC_5_-maleimide (BPM)-labeling assay

The BPM-labeling assay was performed as previously reported [23]. Recombinant Xpo1 was incubated with 4-HNE at 37°C for 1 hour, followed by the addition of Biotin-PEAC_5_-maleimide and further incubation at 37°C for 30 minutes. Protein separation was performed via SDS-PAGE. Thereafter, the proteins obtained were transferred onto a polyvinylidene fluoride (PVDF) membrane or subjected to silver staining with Sil-Best Stain One (Nacalai Tesque). After two washes with Tris-buffered saline with Tween 20 (TBST), the PVDF membrane was blocked with a blocking buffer (Nacalai Tesque) at room temperature for 1 hour. Subsequently, HRP-streptavidin was added and allowed to react with the protein at room temperature for 1 hour. Chemiluminescence was then induced using Chemi-Lumi One Super, and the membrane was visualized using the iBright FL1500 Imaging System (Thermo Fisher Scientific).

### Preparation of nuclear extract

Cells were transfected with an EGFP expression plasmid and cultured in a 10 cm diameter dish. After 2 days, the cells were treated with 10 μg/mL of cycloheximide (CHX) for 30 minutes, followed by 3-hour incubation with 20 μM of 4-HNE. Nuclear extraction was then performed using NE-PER Nuclear and Cytoplasmic Extraction Reagents (Thermo Fisher Scientific). After the protein concentration in the nuclear extract was quantified, 5 μg of the protein extract was used for western blot analysis.

### Protein concentration quantification

The BCA Protein Assay kit (Thermo Fisher Scientific) was used to quantify the protein concentrations in the samples. BSA was used as a standard.

### Polyacrylamide gel electrophoresis (SDS-PAGE)

The gel for electrophoresis was prepared using a 10% TGX FastCast Acrylamide Kit (Bio-Rad), glass plates, and clips, following the manufacturer’s protocol. The samples thus obtained, along with 5% 2-mercaptoethanol (Wako), were mixed with 2× Laemmli Sample Buffer (Bio-Rad) in a 1:1 ratio and incubated at 95°C for 5 minutes. After incubation, the samples were loaded into gel lanes and electrophoresis was conducted using a Mini-PROTEAN Tetra System (Bio-Rad) at 200 V for 30 minutes. The electrophoresis buffer consisted of 1× Tris/Glycine/SDS Buffer (Bio-Rad) diluted with ultrapure water.

### Western blot analysis

Following SDS-PAGE, the proteins were transferred onto a PVDF membrane using a Trans-Blot Turbo system (Bio-Rad). The PVDF membranes were washed twice with TBST and blocked with Blocking one at room temperature for 1 hour. After removing the blocking agent, the membrane was incubated with primary antibodies diluted in TBST (containing 5% blocking one) overnight at 4°C. The next day, the membrane was washed three times with TBST for 5 minutes each and subsequently incubated with secondary antibodies diluted in TBST (containing 5% blocking buffer) at room temperature for 1 hour. After another round of washing with TBST, chemiluminescence was induced using Chemi-Lumi One Super and the membrane was imaged using the iBright FL1500 Imaging System (Thermo Fisher Scientific).

### Silver staining

Silver staining was performed using Sil-Best Stain One (Nacalai Tesque) according to the manufacturer’s protocol.

### Statistical analysis

Graphs are shown as mean ± standard error of the mean (SEM). Statistical analysis was carried out using non-repeated measures analysis of variance (ANOVA) followed by Tukey’s multiple comparison post-hoc test for multiple-group comparisons, or student’s t-test for two-group comparisons, using GraphPad Prism (GraphPad Software Inc.).

## Results

### Accumulation of p62 in the nucleus induced by 4-HNE treatment

**Fig 1:**
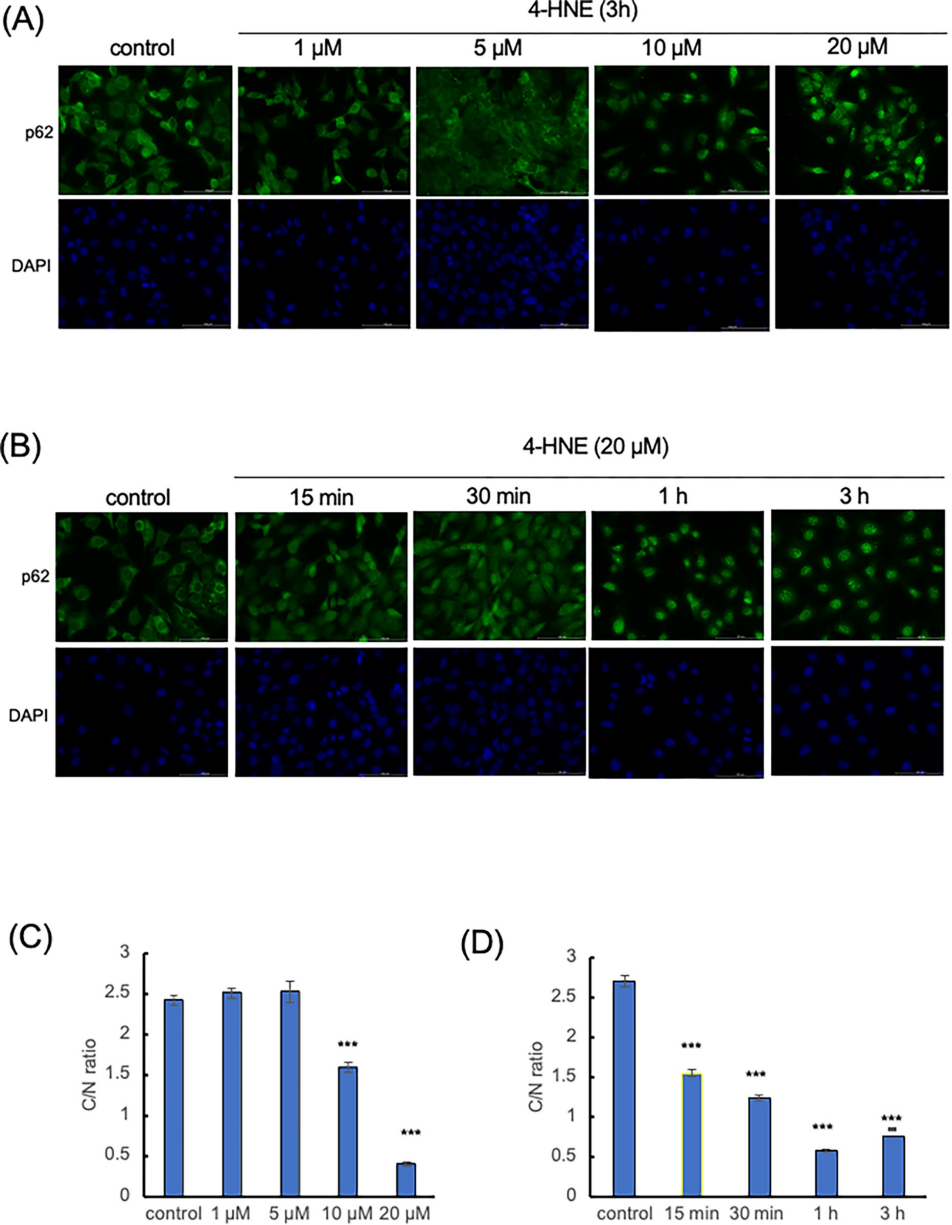
Nuclear accumulation of p62 induced by 4-Hydroxy-Nonenal (4-HNE) treatment. (A) Wild-type mouse embryonic fibroblasts (MEFs) were treated with increasing concentrations of 4-HNE (≥ 10 μM) for 3 hours, and p62 localization was assessed via immunostaining. (B) MEFs were treated with 20 μM 4-HNE for varying durations (15, 30, 60, and 120 minutes) to examine the temporal dynamics of p62 accumulation in the nucleus. DAPI staining was used to visualize nuclei. (C, D) Quantification of p62 fluorescence in the cytoplasm and nucleus was performed using ImageJ, and the cytoplasmic-to-nuclear ratio was calculated for each condition (n = 50 cells per group). Statistical analysis was conducted using one-way analysis of variance (ANOVA) followed by Tukey’s multiple comparisons test, with significant differences indicated (*** *p* < 0.001).

Our study investigated the impact of 4-HNE on p62 localization within wild-type mouse embryonic fibroblasts (WT-MEFs). Immunostaining analysis revealed a significant nuclear accumulation of p62 when WT-MEFs were treated with 4-HNE at concentrations ≥ 10 μM for 3 hours (Fig 1A and 1C). This finding indicates that 4-HNE can induce p62 translocation into the nucleus.

To further elucidate the temporal dynamics of p62 nuclear translocation, WT-MEFs were treated with 20 μM 4-HNE for various time points (15, 30, 60, and 120 minutes). Our results demonstrated that nuclear accumulation of p62 was evident as early as 15 minutes post-treatment (Fig 1B and 1D). The accumulation persisted over time, suggesting a rapid response of p62 to oxidative stress induced by 4-HNE.

These results collectively demonstrate that 4-HNE-induced nuclear accumulation of p62 occurs within a short timeframe, specifically within 15 minutes of treatment, and is concentration-dependent, requiring 4-HNE concentrations of at least 10 μM. This suggests a possible role for nuclear p62 in cellular response mechanisms to oxidative stress at early stages.

### Inhibition of nuclear export by 4-HNE independent of NES modification

To explore the impact of 4-HNE on nuclear export, we constructed vectors encoding pEGFP, pEGFP-NLS (SV40-derived), and pEGFP-NLS-NES (SV40-derived NLS and p62-derived NES), and examined the localization of EGFP in WT-MEFs transfected with these constructs. Under normal conditions, EGFP-NLS-NES was distributed between the cytoplasm and the nucleus, while treatment with leptomycin B (LMB), an Xpo1 inhibitor, resulted in its nuclear accumulation, demonstrating the protein’s ability to shuttle between the nucleus and cytoplasm. This confirmed that EGFP-NLS-NES could be utilized as a model protein to study nuclear-cytoplasmic shuttling.

Upon treating WT-MEFs with 20 μM 4-HNE, we observed nuclear accumulation of EGFP-NLS-NES, suggesting that 4-HNE inhibits NES-dependent nuclear export on a broader scale. Since 4-HNE is known to form stable adducts by reacting with cysteine, histidine, and lysine residues, we considered whether modification of the two lysine residues within the NES might be responsible for inhibiting nuclear export. To further investigate this, we constructed a mutant vector, pEGFP-NLS-NES(mut), in which the NES lysine residues were replaced with serine, and expressed it in MEFs.

**Fig 2:**
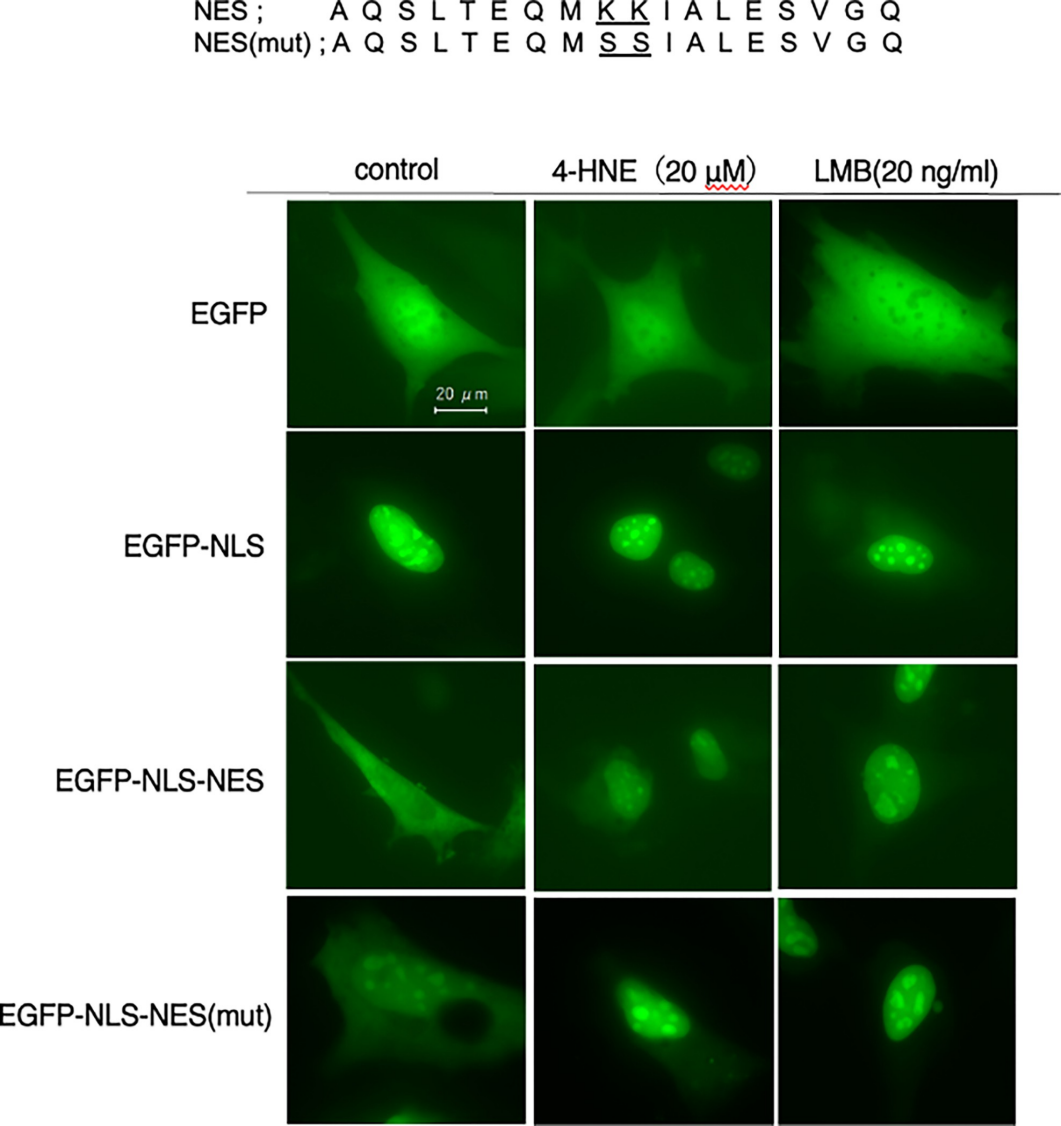
Inhibition of NES-dependent nuclear export of p62 by 4-HNE is independent of NES lysine modification. To examine whether 4-HNE inhibits nuclear export of p62 by modifying NES lysine residues, WT-MEFs were transfected with pEGFP, pEGFP-NLS(SV40-derived), pEGFP-NLS(SV40-derived)-NES(p62-derived), or a mutant pEGFP-NLS-NESmut in which lysines were replaced with serines. After 48 hours, cells were treated with 20 μM 4-HNE or 20 ng/mL leptomycin B (LMB) for 1 hour, and EGFP localization was analyzed. Both 4-HNE and LMB induced nuclear accumulation of EGFP-NLS-NES, including the mutant, indicating that 4-HNE inhibits NES-dependent nuclear export independent of NES lysine modification.

The results showed that, similar to the wild-type construct, EGFP-NLS-NES(mut) also accumulated in the nucleus following treatment with either LMB or 4-HNE (Fig 2). This indicates that the lysine residues within the NES are not direct targets of 4-HNE for inhibiting nuclear export, implying that 4-HNE may act through a different mechanism to block NES-dependent nuclear export.

### Direct binding of 4-HNE to Xpo1

**Fig 3:**
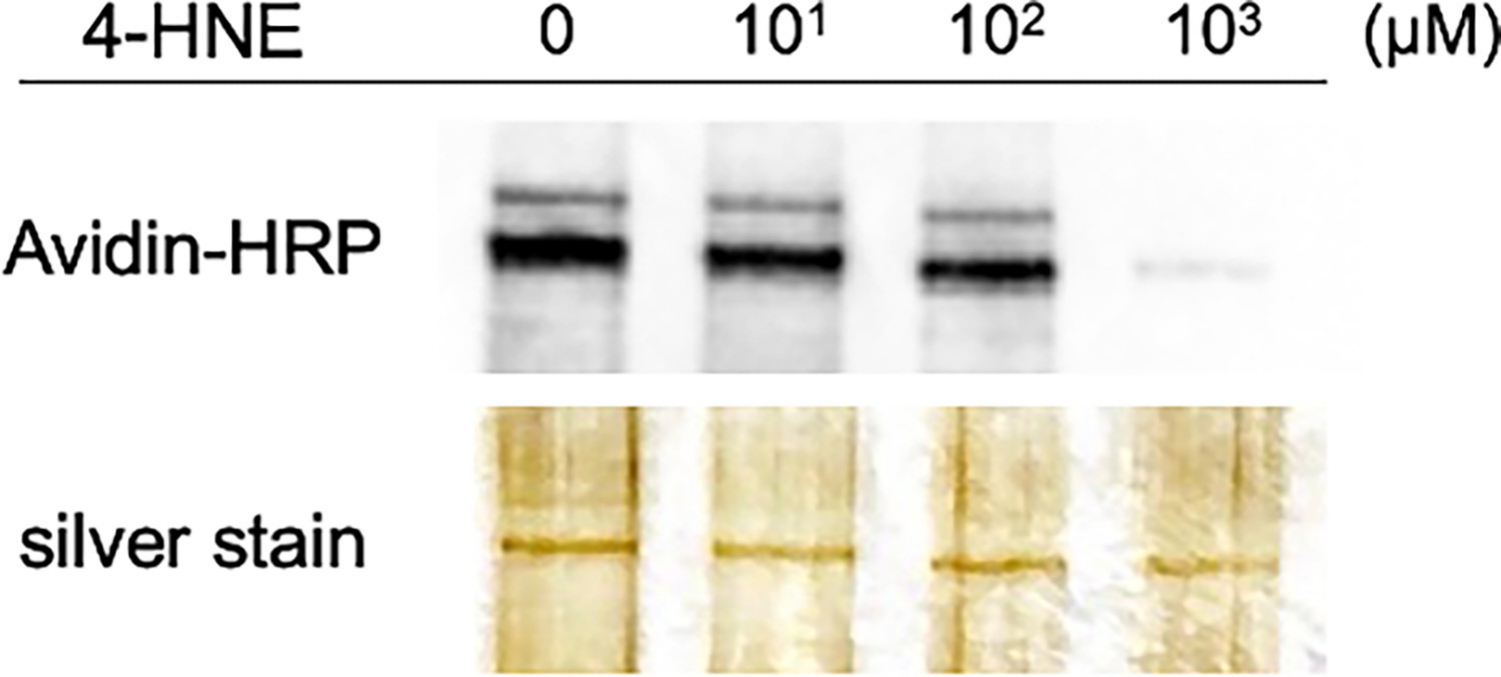
Direct binding of 4-hydroxy-nonenal (4-HNE) to Xpo1. Biotin-PEAC_5_-maleimide (BPM) specifically modifies thiol groups, and if 4-HNE modifies Xpo1, BPM cannot bind to Xpo1, resulting in reduced detection by avidin-horseradish peroxidase (HRP). Recombinant Xpo1 was incubated with 4-HNE at concentrations of 10, 10^2^, or 10^3^ μM, followed by BPM labeling, and subsequently analyzed via electrophoresis. The bands detected by avidin-HRP chemiluminescence shifted towards a lower molecular weight with increasing concentrations of 4-HNE, accompanied by a decrease in band intensity. Importantly, the amount of Xpo1 protein was equal across all samples.

Recombinant Xpo1 was incubated with 4-HNE at concentrations of 10, 10^2^, and 10^3^ μM, and 4-HNE-induced chemical modifications of Xpo1 were assessed using the Biotin-PEAC_5_-maleimide (BPM) method. Biotin-PEAC_5_-maleimide binds to nucleophilic thiols through its electrophilic maleimide group, but it does not react with thiols that have undergone prior electrophilic modification. This property was used to evaluate whether 4-HNE directly interacts with Xpo1. Therefore, if 4-HNE directly modifies Xpo1 electrophilically, it is expected that the biotin-maleimide added later will not react with Xpo1 and will not be detected by avidin-HRP. We observed a concentration-dependent decrease in band intensity with increasing concentrations of 4-HNE, indicating that 4-HNE directly binds to Xpo1 (Fig 3).

### Promotion of EGFP degradation by 4-HNE in the nucleus

**Fig 4:**
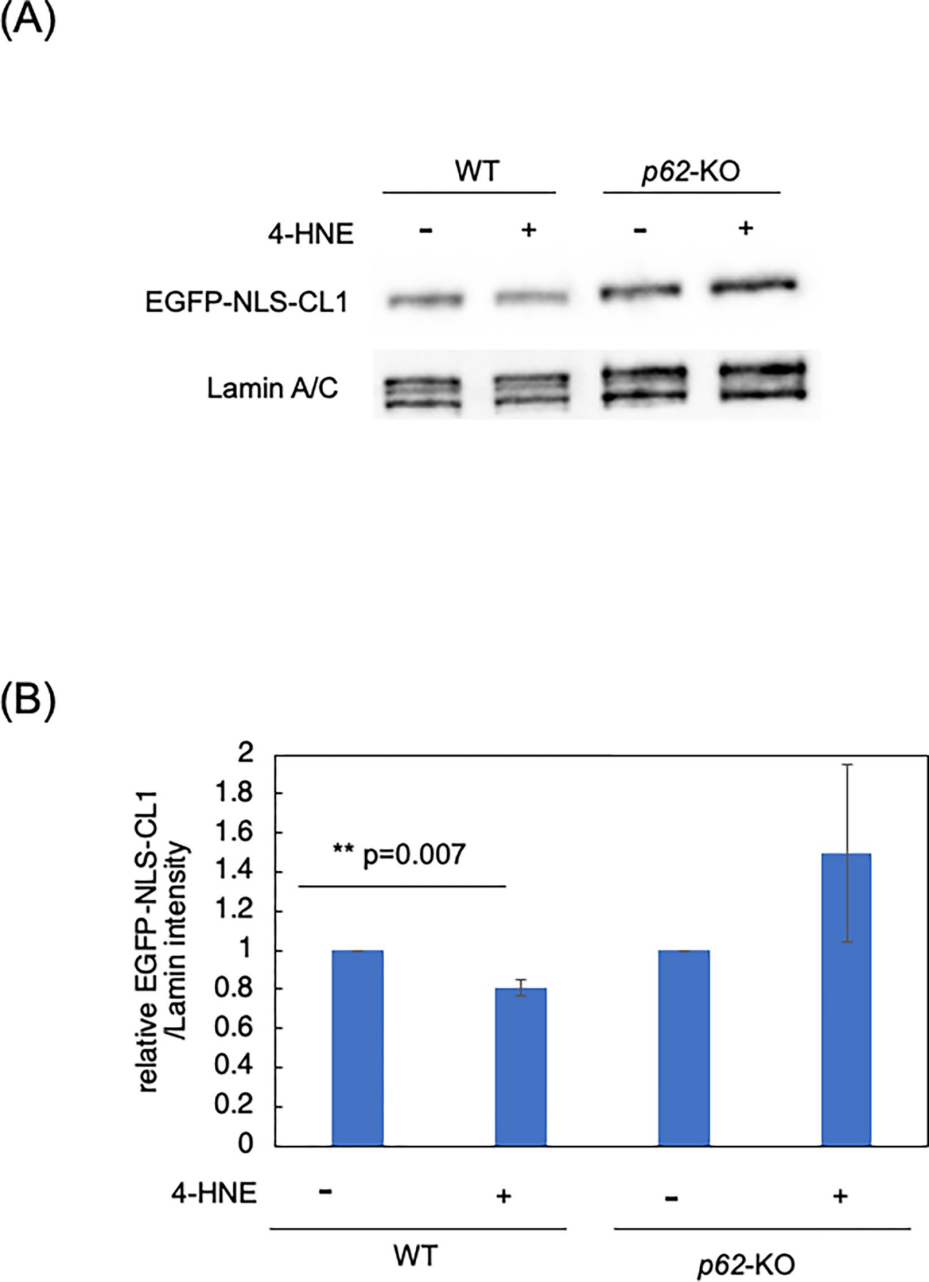
Promotion of EGFP degradation by 4-HNE treatment. (A) Wild type (WT)and *p62*-Knockout (*p62*-KO) mouse embryonic fibroblasts were transfected with the pEGFP-NLS-CL1. EGFP-NLS-CL1 is expected to localize in the nucleus and undertook proteasomal degradation. After 48 hours, the cells were treated with cycloheximide (CHX) for 30 minutes to prevent further supply of newly synthesized EGFP-NLS-CL1. Then, the cells were treated with 20 μM of 4-HNE for 3 hours, nuclear extracts were prepared. 5-μg protein samples were used for western blot analysis to detect EGFP-NLS-CL1 by using anti-EGFP antibody. The experiments were performed in triplicates and representative results are shown. (B) The EGFP-NLS-CL1 band density quantified using ImageJ software. The EGFP-NLS-CL1/Lamin A/C values of 4-HNE-treated samples were calculated relative to the respective 4-HNE-untreated samples. The data shown are the means of three independent experiments. Differences between two groups of 4-HNE-treated and non-treated WT and *p62*-KO cells were analyzed by performing the Student’s *t*-test.

It has been reported that p62 forms droplets via liquid-liquid phase separation in the nucleus and promotes the ubiquitin-proteasome system [20]. Therefore, we investigated the effect of 4-HNE-induced nuclear p62 accumulation on protein degradation activity in the nucleus by using EGFP-NLS-CL1. CL1 is known to a degron for 26S proteasome, act as a signal for proteasome-dependent degradation in both yeast and mammals [24]. Two days after pEGFP-NLS-CL1 transfection into WT or *p62*-KO MEFs, the cells were treated with CHX at 10 μg/mL for 30 minutes to prevent further supply of new EGFP-NLS-CL1. Then, the cells were treated with 20 μM 4-HNE for 3 hours. As a result, western blot analysis of nuclear extracts showed that in *p62*-KO cells, EGFP degradation was unaffected by 4-HNE, whereas in WT cells, the amount of EGFP decreased by approximately 20% compared with that observed in the untreated cells (Fig 4(A) and 4(B)).

## Discussion

p62 is involved in selective autophagy in the cytoplasm via its interaction with LC3 [16]. However, it has been reported that in the nucleus, it form droplets and thus, contributes to the efficiency of the UPS [20]. However, several uncertainties exist regarding the mechanisms and physiological significance of its nuclear translocation.

In this study, we demonstrated that treatment with 4-HNE at concentrations ≥ 10 μM induced nuclear accumulation of p62 within 15 minutes. To understand the underlying mechanism, we used the model protein EGFP-NLS-NES, which is exported from the nucleus in an Xpo1-dependent manner. Under normal culture conditions, EGFP-NLS-NES was distributed in both the cytoplasm and nucleus, but treatment with leptomycin B (LMB), a specific inhibitor of Xpo1, resulted in its nuclear localization. This indicates that EGFP-NLS-NES shuttles between the cytoplasm and nucleus in an Xpo1-dependent manner.

Similarly, treatment with 4-HNE induced nuclear localization of EGFP-NLS-NES, suggesting that 4-HNE inhibits Xpo1-mediated nuclear export. Furthermore, since the NES contains amino acids that are potential targets of 4-HNE, we used a mutant EGFP-NLS-NES, where the Lys residue was replaced by Ser, to examine the effect of 4-HNE. We found that even the mutant protein accumulated in the nucleus in response to 4-HNE, similar to the effects observed with LMB treatment.

Using the BPM method, we also confirmed that 4-HNE directly binds to and oxidatively modifies Xpo1. Taken together, these results strongly suggest that 4-HNE-induced nuclear accumulation of p62 is associated with the inhibition of Xpo1 function through oxidative modification. This indicates that the effect of 4-HNE on nuclear export is not unique to p62 but extends to other Xpo1-dependent cargo proteins, highlighting a broader impact of 4-HNE on nuclear-cytoplasmic shuttling processes.

We also employed EGFP-NLS-CL1 as a model substrate that is degraded by the UPS in the nucleus and demonstrated that 4-HNE enhanced the UPS in the nucleus in a p62-dependent manner. This finding aligns with the results of previous studies, which suggested that p62, via liquid-liquid phase separation in the nucleus, recruits factors involved in the UPS, thereby contributing to efficient protein degradation [20].

Although 4-HNE is known to exert various physiological effects [25–27], this study introduces a novel inhibitory effect on Xpo1. 4-HNE is an electrophilic substance that induces oxidative modifications in various proteins. Keap1, a sensor protein for electrophilic substances, undergoes 4-HNE-induced modification, activates transcription factor Nrf2, and contributes to enhancing antioxidant and xenobiotic metabolic activities [28]. Further, under 4-HNE treatment, abnormal proteins with modifications accumulate in cells [29] and must therefore be removed by molecular chaperones and protein degradation systems to ensure cellular homeostasis. Our results suggested the existence of a potential axis involving 4-HNE-Xpo1 inhibition-p62 nuclear accumulation-UPS activation as a response system for the quality control of nuclear proteins in response to 4-HNE.

An important future challenge will be to elucidate the molecular mechanism by which 4-HNE inhibits Xpo1. Compounds like LMB and clinically utilized anti-cancer agents, selective inhibitor of nuclear export (SINE), target Cys528 in Xpo1 [30]. Considering that 4-HNE has the potential to modify cysteine residues, a similar mechanism is plausible. Further analysis of its mode of Xpo1 inhibition could contribute to the development of novel Xpo1 inhibitors.

In conclusion, this study highlights the possibility that 4-HNE dramatically alters the localization of p62 in the nucleus by inhibiting Xpo1. Thus, it contributes to nuclear protein degradation. Further investigations are needed to explore the regulatory mechanisms of p62 cellular localization and the physiological significance of nuclear-cytoplasmic shuttling.

## Supporting information

S1 Raw images Raw images of Figs 3 and 4. (PDF)

S1 Raw data (XLSX)
