# Supplementary material for: 4-Hydroxy-2-nonenal causes nuclear accumulation of p62 by inhibiting Xpo1 and promoting the proteolytic pathway in the nucleus
Source: PLoS One. 2025 Feb 3;20(2):e0316558. doi: 10.1371/journal.pone.0316558 (PMC11790078; doi:10.1371/journal.pone.0316558)
Supplement: S1 Raw images — (PDF) [file pone.0316558.s001.pdf]

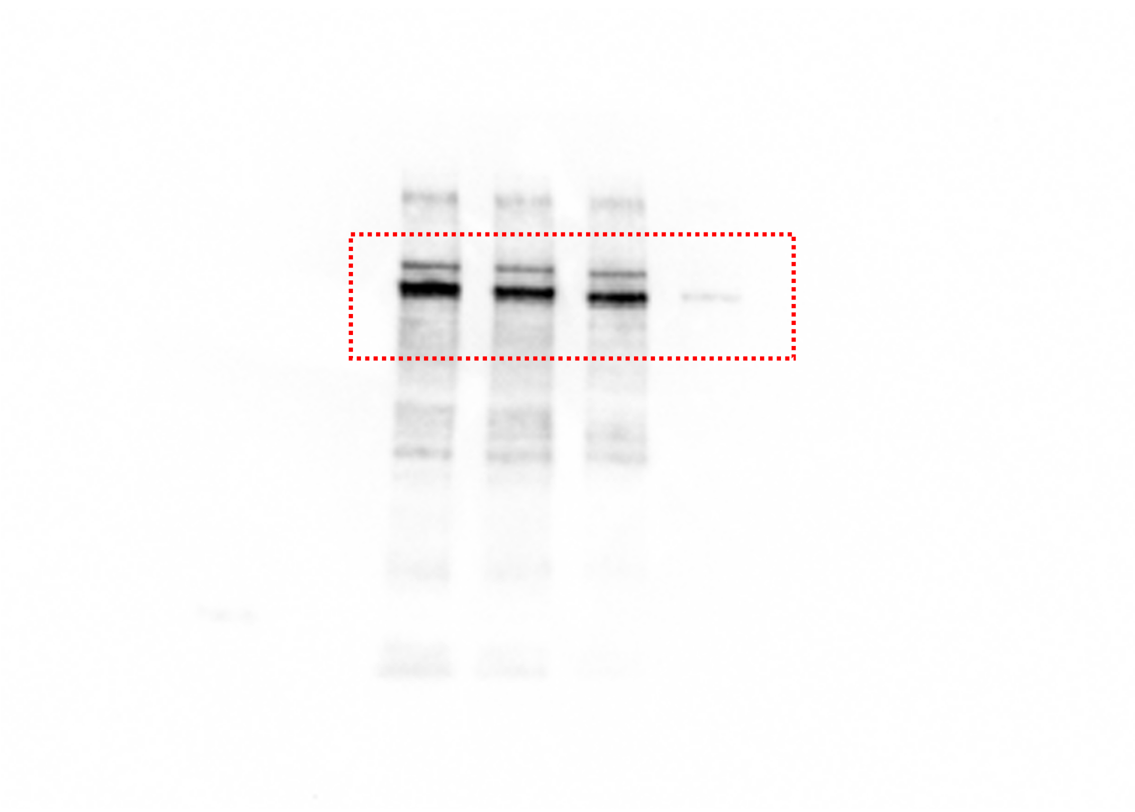

Fig. S1
The original image shown in Fig. 3.
Lane order: .0, 10^1, 10^2, 10^3 μM of the 4-HNE concentration.

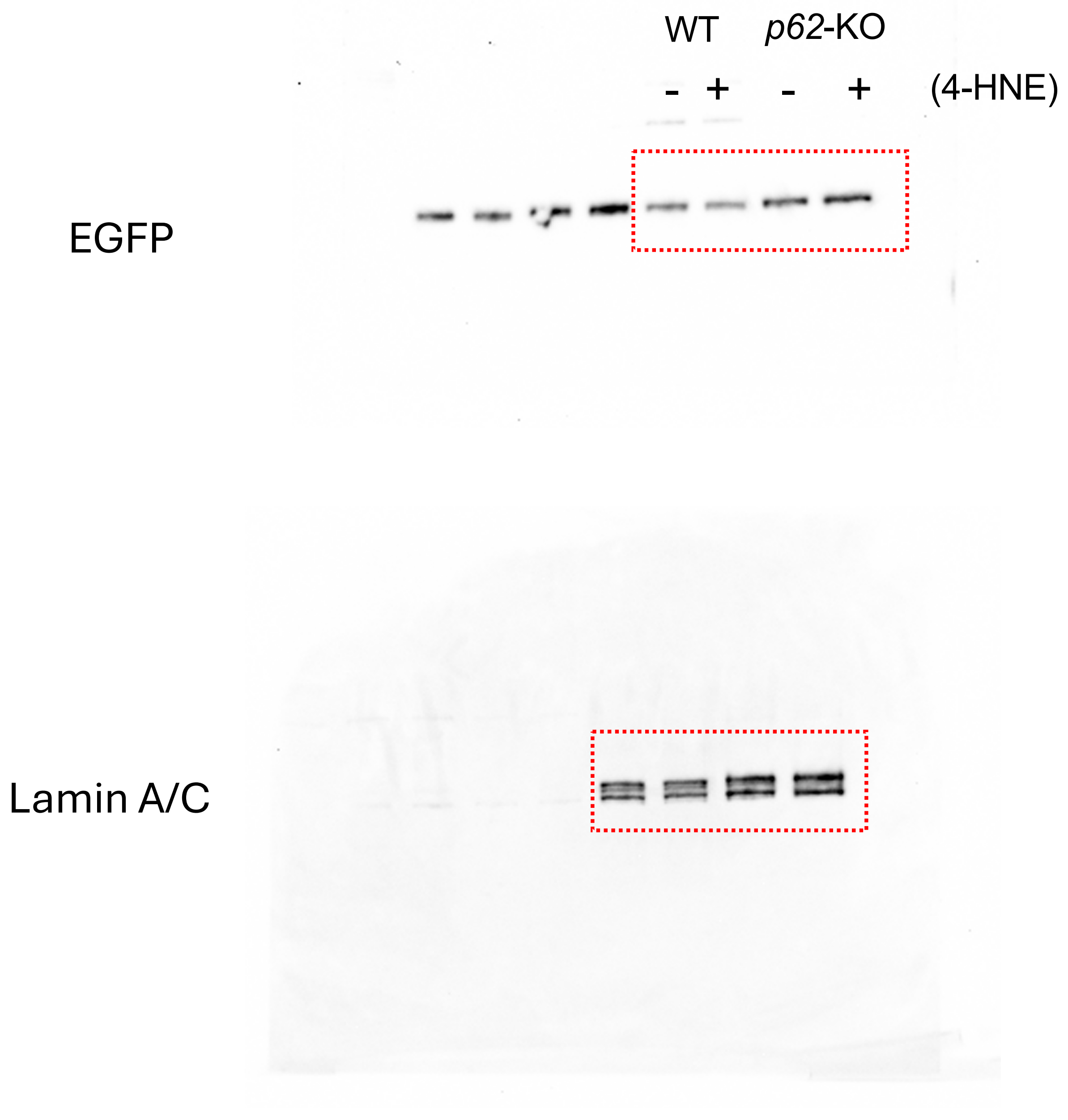

Fig. S2
The original images shown in Fig. 4.
The four lanes on the left are cytoplasmic fractions of the same samples (same order as nuclear fractions).
